# Second Language Processing Shows Increased Native-Like Neural Responses after Months of No Exposure

**DOI:** 10.1371/journal.pone.0032974

**Published:** 2012-03-28

**Authors:** Kara Morgan-Short, Ingrid Finger, Sarah Grey, Michael T. Ullman

**Affiliations:** 1 Department of Hispanic and Italian Studies and Department of Psychology, University of Illinois at Chicago, Chicago, Illinois, United States of America; 2 Brain and Language Lab, Georgetown University, Washington, D.C., United States of America; 3 Department of Modern Languages, Federal University of Rio Grande do Sul, Porto Alegre, Brazil; University Of Cambridge, United Kingdom

## Abstract

Although learning a second language (L2) as an adult is notoriously difficult, research has shown that adults can indeed attain native language-like brain processing and high proficiency levels. However, it is important to then *retain* what has been attained, even in the absence of continued exposure to the L2—particularly since periods of minimal or no L2 exposure are common. This event-related potential (ERP) study of an artificial language tested performance and neural processing following a substantial period of no exposure. Adults learned to speak and comprehend the artificial language to high proficiency with either explicit, classroom-like, or implicit, immersion-like training, and then underwent several months of no exposure to the language. Surprisingly, proficiency did not decrease during this delay. Instead, it remained unchanged, and there was an *increase* in native-like neural processing of syntax, as evidenced by several ERP changes—including earlier, more reliable, and more left-lateralized anterior negativities, and more robust P600s, in response to word-order violations. Moreover, *both* the explicitly and implicitly trained groups showed increased native-like ERP patterns over the delay, indicating that such changes can hold independently of L2 training type. The results demonstrate that substantial periods with no L2 exposure are not necessarily detrimental. Rather, benefits may ensue from such periods of time even when there is no L2 exposure. Interestingly, both before and after the delay the implicitly trained group showed more native-like processing than the explicitly trained group, indicating that type of training *also* affects the attainment of native-like processing in the brain. Overall, the findings may be largely explained by a combination of forgetting and consolidation in declarative and procedural memory, on which L2 grammar learning appears to depend. The study has a range of implications, and suggests a research program with potentially important consequences for second language acquisition and related fields.

## Introduction

Research on adult-learned second language (L2) has provided considerable insight into the neurocognitive mechanisms underlying the learning and processing of L2 grammar [1]–[11]. Of interest here, studies suggest that, despite the difficulties in acquiring L2 grammar, adult learners *can* approximate native-like levels of use and neurocognitive processing [12]–[15]. However, it is not enough to have attained such native-like levels. Crucially, it is also desirable to *retain* them, even in the absence of continued practice or exposure to the L2. In fact, substantial periods (months to years) of limited or no exposure following L2 training are not uncommon, and may even be the norm [16]. Such a scenario may be found in different situations, including when one studies a language in a classroom and then stops taking classes [17], [18] and when one is immersed in a foreign language setting and then moves away [19]. In the present study, we examine the outcomes of such a period of no exposure on the neurocognition of L2 grammar: that is, whether a substantial period of no exposure leads to decreased proficiency and/or less native-like neural processes (“use it or lose it” [20]), no such changes, or perhaps whether even higher proficiency and/or more native-like processing may be observed. Additionally, we test whether any such outcomes might vary as a function of the type of L2 training, in particular between classroom-like and immersion-like contexts.

### Previous Research

We are aware of six studies designed to investigate the effects of a substantial period of limited exposure following adult L2 training [17], [18], [21]–[24], all of which were restricted to the examination of behavioral (performance) outcomes. (Note that we do not consider case studies, purely observational data, or research on L2s acquired by children; for a comprehensive review, see [16]). The six studies tested L2 learners after periods of 1 month to 50 years of limited L2 exposure, mainly on general language skills [17], [18], [21], [23], [24], though also on more specific paradigms meant to target aspects of grammatical abilities [17], [18] or lexical abilities [22], [23]. These language measures were compared in most studies to the same measures in a different set of subjects who had not experienced a period of limited exposure [17], [18], [22], [23], or to retrospective ratings of the same subjects [21], with only one longitudinal study testing the same subjects before and after a period of limited exposure [24]. Across the studies, the periods of limited exposure followed either classroom training [17], [18], [22], [24] or mixed classroom and immersion training [21], [23], [24]. The training lasted varying lengths of time, apparently usually in the range of a few years, and resulted in seemingly varying proficiency levels (though not directly measured, except by [24]) prior to the period of limited exposure.

Overall, the results of the six studies have been taken to suggest the following. A period of limited exposure generally leads to attrition (loss) of L2 performance or knowledge [17], [18], [21], [23]. Such loss has been observed after as little as a few months of limited exposure, e.g., after a 1–7 month [23] or 6 month delay [21], as well as after 2 years [18], though in one case it was observed only by 3–5 years, and not earlier [17]. Although attrition may take place within the first few years, some studies suggest that it then appears to level off, with no further losses occurring [17], [18]. Higher levels of proficiency (or exposure) may be associated with less attrition [17], [18], [21], [23] or even with no observed losses [21]. Moreover, one study found no changes at all in performance, across proficiency levels, after either 2 or 4 years of limited exposure [22]. Finally, in some cases a *gain* in performance has been observed: after 1.5 years of limited exposure in one study, particularly for L2 learners with immersion as well as classroom training [24], and in another study after 2 years, though only for some abilities, such as listening and reading comprehension [18]. It remains unclear what might explain such gains, which have been attributed to motivation and to L2 experience during the period of ostensibly limited exposure [24], or to factors related to general maturation, cognitive development, or continued academic training [18].

Thus, although most studies have reported L2 attrition following a period of limited L2 exposure, the picture is still mixed, and the effects of such periods are still not well understood. This lack of clarity is due both to gaps in the literature and to confounds and other methodological weaknesses in previous studies. First, it is important to emphasize that there has still been very little research examining the effects of limited or no L2 exposure. Second, all such studies have focused on changes in *performance* (e.g., proficiency) after periods of limited exposure, and have largely ignored potential changes in the underlying processing or computational *mechanisms*. Third, all previous research has been restricted to the use of behavioral rather than neural measures. Thus it is still unknown whether or how the neural substrates of an L2 might change following a period of limited or no exposure. Importantly, such neural changes could take place even in the absence of observed behavioral changes, and could shed light on any changes in the L2 processing mechanisms. Fourth, in all six previous studies subjects had at least some L2 exposure during the period of ostensibly limited exposure, and only two studies seem to have attempted to control for this factor [17], [18]. In fact, in at least one study in which gains were observed, the authors attributed these changes to L2 exposure during this period [24]. Thus it remains unclear to what extent any observed changes are due to the time lag or to continuing exposure. Fifth, the lack of longitudinal designs (other than [24]) suggests caution in interpreting previous findings, particularly since various factors that may affect language (e.g., age, education, handedness, sex [25]–[27]) were not controlled for or matched between the subjects, who had experienced a period of limited exposure, and the control subjects, who had not. Indeed, in some cases not even L2-related factors were adequately controlled for, such as the amount (and type) of L2 exposure during training [17], [23]. Sixth, in previous studies, subjects tested after the period of limited exposure differed from controls not only in the period itself, but also in the recency of their exposure to the L2, given that only the subjects in the control condition had had clear recent contact with the language. In other words, the lack of any “warm-up” session following the period of limited exposure confounds the results from most previous studies, thus precluding clear conclusions regarding the impact of such a period. In fact, the only study that did have some warm-up [24] (which was also the only longitudinal study) did not report attrition, but rather no changes in performance as well as gains after the delay, suggesting that the inclusion of a warm-up period might significantly affect the outcomes of studies of limited or no exposure. Seventh, although some studies claimed that their subjects had reached high proficiency [23] or very high proficiency [18], [22] prior to the period of limited exposure, proficiency or other aspects of performance were not directly measured in these studies, but were rather inferred indirectly from the control group. This suggests caution in interpreting these findings regarding the effects of limited L2 exposure following the attainment of high proficiency. Eighth, while few studies have examined the effects of periods of limited exposure on L2, even fewer have investigated such effects specifically on grammar [16]. Moreover, those studies that have done so [17], [18] have examined grammar from a traditional language instruction perspective rather than from a psycholinguistic approach, making it more difficult to draw conclusions about any changes in the knowledge or processing of grammar. Finally, no studies have examined or isolated the effects of classroom vs. immersion training on the outcome of a period of limited exposure, even though some of the findings hint that immersion might lead to advantages as compared to classroom training following such a period [24].

The contrast between explicit, classroom-like, and implicit, more immersion-like training is important in the present study. This contrast is motivated by a considerable body of behavioral research that has previously examined the effectiveness of explicit versus implicit training on L2 learning [28]–[30]. Explicit treatments in these studies provide learners with information about the grammar rules or direct them to search for rules, whereas implicit treatments are designed to engage learners with the target language, but do not provide explicit information or direction to search for rules [28].

The relative efficacy of the two types of treatment remains unclear. On the one hand, a recent meta-analysis of 30 studies by Spada and Tomita [29] found that explicit treatments were more effective than implicit ones on L2 development, not only immediately after training but also after a delay (typically less than a few weeks). This result echoes conclusions from a previous meta-analysis based on earlier studies [28]. On the other hand, these putative advantages for explicit treatments are compromised by several issues [31]. Perhaps most problematically, the designs of previous studies likely favored the outcomes of explicit treatments [28], [32], [33]. For example, these treatments often provided learners with more input and/or more time-on-task than the implicit treatments. Moreover, the assessment tasks themselves generally focused on explicit knowledge, further biasing the outcome. Another issue is that the subjects in previous studies examining explicit vs. implicit training had not reached high L2 proficiency, either before or even after the treatment [34]–[36]. Thus, the efficacy of one treatment type over another at attaining, let alone at subsequently retaining, high proficiency remains very much in question. Finally, in previous studies any delays were quite short (see above), and moreover, additional exposure to the L2 often occurred during this period. Therefore it remains unclear whether substantial periods of no exposure yield the same or different performance (let alone neural processing) outcomes for explicit and implicit training.

The present study was designed to address some of these gaps and issues. We used Event-Related Potentials (ERPs) together with behavioral measures to examine the effects of a period of no L2 exposure on the neurocognition of L2 grammatical (syntactic) processing in adult learners. Subjects were tested at two time points in a longitudinal (within-subjects) design: first, immediately after they learned the language to high proficiency, and second, after a period of several months during which they had no exposure at all to the L2 (achieved by virtue of our artificial language paradigm). Immediately prior to both test sessions, subjects were given equivalent brief warm-up practice sessions to avoid recency confounds (see above). Additionally, in a between-subjects design, we compared these before-and-after effects between two L2 training groups, one who received classroom-like (explicit) training, while the other received immersion-like (implicit) training, thus enabling us to distinguish any differential effects of the type of training on the outcome of a period of no L2 exposure.

### Event-Related Potentials and Language

As we have seen, previous research examining the effects of a period of limited or no exposure has been restricted to the examination of proficiency and related behavioral outcomes. Although such measures of L2 attainment can reveal how *well* an L2 is learned, they cannot easily tell us what processing or computational mechanisms, let alone what neural systems, underlie its learning and use. Event-Related Potentials may be the best method for achieving these goals. ERPs reflect real-time scalp-recorded electrophysiological brain activity of cognitive processes that are time-locked to the presentation of target stimuli. ERPs together with behavioral data provide complementary and synergistic measures, and improve the likelihood of detecting differences between conditions or groups. Indeed, ERPs can be sensitive to effects that are not found with behavioral measures, including in L2 studies [37], [38]. Unlike other neuroimaging techniques (fMRI, MEG), ERP research has revealed a set of widely-studied language-related activation patterns (“ERP components”) in first language, whose characteristics and associated processing mechanisms are reasonably well understood (see just below). Importantly, these components provide a clear frame of reference for examining L2 processing, including in studies of artificial languages [31], [39], [40]. Finally, unlike hemodynamic imaging methods like fMRI, ERPs provide excellent temporal resolution, allowing one to examine the actual time course of processing.

ERP research has shown that in first language (L1), lexical/semantic anomalies elicit an N400 (e.g., I drink coffee with milk and *spit, where * marks the violation) [41]. This negative waveform typically shows a central/posterior bilateral distribution, and peaks about 400 ms post-stimulus. N400s reflect aspects of lexical/semantic processing, and may depend on the declarative memory brain system [10], [42], [43]. In contrast, disruptions of rule-governed (morpho)syntactic processing, such as violations of word order (phrase structure), which are examined in the present study, frequently yield two components in L1. First, they can, though do not always [44], [45], elicit early but sometimes continuing left-to-bilateral anterior negativities that can extend to central sites [46]–[49]. The initial portions of these negativities (150–500 ms), which are often but not always left lateralized, seem to reflect aspects of rule-governed structure-building [50]–[52], and have been posited to depend on the procedural memory brain system that appears to underlie rule-governed compositional aspects of grammar [10], [53]. Later portions of the negativities, beginning around 500 or 600 ms, generally show bilateral distributions [14], [47], [51], [54], [55]. It remains unclear whether the earlier and later anterior negativities represent the same or distinct components [56]. It has alternatively been suggested that the later anterior negativities constitute continuations of the earlier ones [49], [56], or that they may reflect a different process, in particular, one related to increased working memory demands [54]. Interestingly, anterior negativities that are less left-lateralized (more bilateral), and that spread into central sites and are temporally more extended (i.e., that also occur in later time windows) may be associated with lower L1 proficiency [49]. Crucially, regardless of whether the earlier and later anterior negativities represent the same or distinct components, both are frequently observed in response to (morpho)syntactic violations in L1, and thus both appear to be representative of native-like processing [48], [56], [57]. Second, (morpho)syntactic disruptions also usually elicit P600s: late (600 ms) centro-parietal positivities [58], [59] that have been linked to controlled (conscious) processing, syntactic integration, and structural reanalysis [43], [51], [58]–[60]. Finally, the anterior negativity/P600 biphasic pattern may be particularly characteristic of native-speaker processing of (morpho)syntactic violations [43], [47], [48], [52], [56].

In L2, lexical/semantic violations elicit N400s at both low and high L2 proficiency, though sometimes at reduced amplitudes and/or with a delayed time-course as compared to L1 [5], [10], [37], [61]. (Note that L2 proficiency and exposure are usually correlated and are difficult to tease apart; for simplicity, in this paper we usually refer only to proficiency levels rather than to both proficiency and exposure; also see Discussion.) Violations of (morpho)syntax do not usually elicit anterior negativities at low L2 proficiency. Rather, at low L2 proficiency such violations tend to elicit either no component [62], [63] or N400 or N400-like responses [31], [40], [64], [65], suggesting a compensatory role for lexical/semantic processes, and possibly declarative memory, at low proficiency. In contrast, at high L2 proficiency (morpho)syntactic violations often elicit anterior negativities. These are generally found in earlier time windows [5], [14], [31], [63], [66], [ but see 67], though they often extend to later ones [14], [31], [66]. The anterior negativities in these studies have generally been bilaterally distributed and may include more central sites [14], [66], possibly due to lower L2 proficiency [5]. In L2, (morpho)syntactic violations generally also elicit P600s, particularly but not only at higher L2 proficiency [5], [14], [31], [40], [64], [65]. Finally, in some studies of high L2 proficiency, including for artificial languages, (morpho)syntactic disruptions elicit an L1-like anterior negativity/P600 biphasic response [5], [14], [15], .

In sum, ERP research suggests that while the neurocognition of lexical/semantic processing is similar in L1 and L2, the neurocognitive processes underlying L2 (morpho)syntax depend at least in part on the learner's level of proficiency (or exposure), with higher proficiency levels associated with greater L1-like processing. However, to our knowledge no ERP (or other neurocognitive) research has investigated what takes place *after* high proficiency has been reached, following a substantial period of non-exposure to the L2. In fact, previous studies examining the neurocognition of low or high proficiency L2 in natural languages have essentially ignored this issue. Since it seems possible or even likely that many of their subjects experienced substantial periods of limited or no L2 exposure, it is not clear to what extent the results from these studies might be attributed to proficiency levels and/or to periods of limited or no exposure (also see Discussion). Finally, although recent work (which forms the basis of the present study) has examined whether explicit or implicit training might be better for achieving native-like neurocognitive processing [31], [40], no research has investigated whether or how these training types might differentially impact the neurocognitive effects of a period of non-exposure to the L2.

### An Artificial Language Approach

To provide an empirical answer to these questions, the present study complemented its use of ERPs with an artificial language paradigm. The use of artificial linguistic systems, which include both artificial languages and artificial grammars, is a well-established method in both the fields of Second Language Acquisition (SLA) [68]–[71] and cognitive (neuro)science [39], [72]–[78]. Artificial linguistic systems crucially allow for the design of experiments that have precise control over the variables of interest, which may be difficult if not impossible to control in natural language studies. Artificial *languages* are more natural-language-like than artificial *grammars*. Like natural languages (but unlike artificial grammars), artificial languages contain a lexicon and grammatical rules that preserve form-meaning relationships among items. The lexicon is typically composed of novel (made-up) words, while the rules are consistent with the rules found in natural languages. The language is generally presented purely auditorily. Thus artificial languages, which subjects can learn to actually speak and comprehend, are simplified models of natural languages. Moreover, they have been found to elicit the same neural patterns observed in natural language studies, further validating their utility in language learning and processing research [31], [39], [40]. However, unlike natural languages, artificial languages can be learned to high proficiency in a matter of hours to days, providing the ability to examine the learning trajectory longitudinally to high proficiency, and to fully control L2 training conditions in the laboratory. Indeed, like other artificial linguistic systems, artificial languages allow for control over multiple variables that are difficult if not impossible to fully control in L2 research, including (dis)similarity to the L1 and the amount of exposure both during and following training, as well as the type of training itself, such as explicit, classroom-like and implicit, immersion-like treatments. Thus, like other simplified models of complex systems in science, using an artificial language provides the means to rapidly and reliably (avoiding confounds) identify the factors or mechanisms of interest. And as with other such models, one can subsequently focus on directly testing these already-identified factors and mechanisms in the slower and more difficult examination of the full complex system of interest, in this case natural language. Thus artificial languages constitute a “test tube” model of the study of natural language [31], [39], [79].

The only study we are aware of that has compared the effects of explicit and implicit training on the attainment of high L2 proficiency has in fact done so with an artificial language [31]. This study, which examined ERPs as well as behavioral outcomes, observed a different pattern from studies investigating the effects of explicit vs. implicit training at lower levels of proficiency. In contrast to the majority of previous research (see above), no particular advantages were observed for explicit training on behavioral measures. Moreover, more native-like ERP patterns were found in the implicitly than explicitly trained group at high proficiency. It is this study that forms the basis of the present one, in that it is a subset of these subjects who were tested after a subsequent period of no exposure.

### The Present Study

In brief, the present study examined the effects of a substantial period of no L2 exposure on the neurocognition of syntactic (word order) processing in subjects who had attained high L2 proficiency under either explicit, classroom-like or implicit, immersion-like training conditions. Specifically, adult native English-speaking monolingual subjects learned to speak and understand the artificial language Brocanto2 (which has different syntactic properties from English) to high proficiency following either explicit or implicit training. ERPs and acceptability judgments for correct Brocanto2 sentences, and for sentences with word order violations, were each acquired twice, once immediately post-training (“end of training”), at which point high proficiency had been reached, and then again following a several month period of no exposure to the language (“retention”). Immediately prior to both of these behavioral/ERP assessments, subjects were given a brief warm-up session. Based on the fact that the preponderance of previous studies have shown L2 attrition following a period of limited exposure, even after a few months, we expected a decrease in performance (proficiency) between end of training and retention, though the strength of this prediction was modulated by the absence of post-delay warm-up sessions in previous studies. Given the lack of previous studies examining the neural processing effects of such a period, we had no specific predictions regarding possible changes to the ERP pattern.

## Methods

### Subjects

We tested 21 adults 3 to 6 months after they had learned Brocanto2 in a prior experiment (the original study), in which subjects had been trained on the artificial language under either explicit or implicit conditions [31], [40]. All participants were right-handed [80], had no known developmental, neurological or psychiatric disorders, and had normal or corrected hearing and vision. All were native speakers of English who were not fluent in any other language. Because the artificial language was structurally similar to Romance languages, all participants had limited exposure to Romance languages (no more than three years of classroom exposure to any Romance language, and no more than two weeks of immersion in a Romance language environment). Of the 21 subjects, two were excluded from analysis, one due to a large number of artifacts in the ERP data, and the other due to a technical problem with the data file. In the original study, these 19 subjects had been randomly assigned to two training groups: 10 had learned Brocanto2 under the explicit training condition, while 9 had learned it under the implicit training condition.

These 10 explicitly and 9 implicitly trained subjects did not differ in sex (explicit: 5 females out of the 10 subjects, or 50%; implicit: 4 out of the 9 subjects, or 44%) or in the number of participants who returned from the original study for testing 3 to 6 months later (explicit: 10 out of 16, or 62.5%; implicit: 9 out of 14, or 64.3%). The explicitly and implicitly trained participants also did not differ (unpaired *t*-tests, *p*s>0.05) on: the number of days between completion of the original study and testing in the present study (explicit: *M* = 158.20, *SD* = 31.38, range = 105–206; implicit: *M* = 156.67, *SD* = 33.76, range = 92–197); age (explicit: *M* = 24.40 years, *SD* = 4.33; implicit: *M* = 27.00 years, *SD* = 5.70); years of education (explicit: *M* = 16.10, *SD* = 3.07; implicit: *M* = 17.44, *SD* = 1.94); age of first exposure either to Romance languages (explicit: *M* = 11.33, *SD* = 1.53; implicit: *M* = 12.00, *SD* = 0.00) or to any other second language (explicit: *M* = 12.71, *SD* = 1.97; implicit: *M* = 14.38, *SD* = 1.92); or years of exposure to either Romance languages (explicit: *M* = 1.68, *SD* = 1.37; implicit: *M* = 2.33, *SD* = 1.00) or to any other non-native language (explicit: *M* = 1.38 years, *SD* = 2.24; implicit: *M* = 2.83 years, *SD* = 3.93). All subjects gave written informed consent and received monetary compensation for their participation, which was approved by the Georgetown University IRB.

### The Artificial Language

All participants had learned the artificial language Brocanto2 in the original study [31], [40]. Brocanto2 follows universal requirements of natural languages, is fully productive, and can be actually spoken and comprehended. It is based on the artificial language Brocanto. Both Brocanto and Brocanto2 have elicited natural language brain patterns in ERP and/or fMRI studies [31], [39], [40], [73].

**Figure 1:**
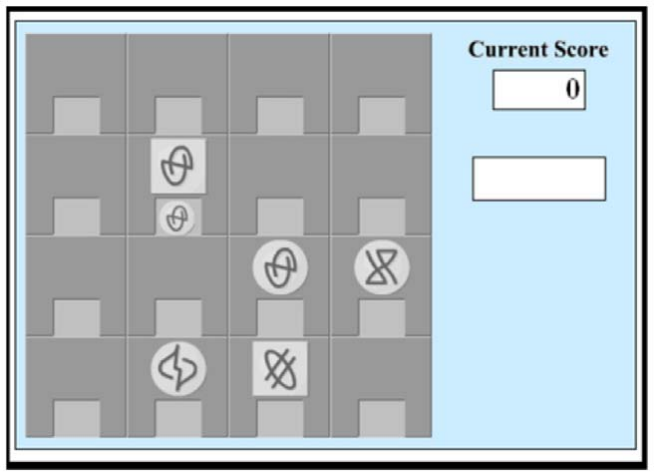
Computer-based game board. Game tokens are represented by visual symbols, which correspond to nouns in Brocanto2. The tokens can further be distinguished by their background shape–square or round–each of which corresponds to a Brocanto2 adjective. Players can move, swap, capture, and release tokens, with each of these actions corresponding to Brocanto2 verbs, as well as move them either horizontally or vertically (corresponding to Brocanto2 adverbs).

The lexicon of Brocanto2 consists of 13 novel words with English pronunciation and phonotactics: 1 article (*l-*), marked for gender (masculine *li*; feminine *lu*); 2 adjectives (*trois*-, *neim*-), each marked for gender (masculine *troise/neime*; feminine *troiso/neimo*); 4 nouns (*pleck, neep, blom, vode*), two of which are masculine and two feminine (the nouns are not overtly marked for gender, but their articles and adjectives must agree with them); 4 verbs (*klin, nim, yab, praz*); and 2 adverbs (*noyka, zayma*). (Note that since Brocanto2 is presented solely auditorily, the orthographic representations presented here are provided only for the reader.) In contrast to English, articles and adjectives in Brocanto2 are post-nominal (i.e., noun-[adjective]-determiner) and morphologically marked so as to agree in gender with the noun to which they refer. Also unlike English, Brocanto2 sentences have a fixed subject-object-verb word order and have no morphological features on the verb. Adverbs, when used, immediately follow the verb. All the grammatical features of Brocanto2 are found in natural languages, such as Supyire (spoken in Mali), which has subject-object-verb word order, grammatical gender agreement, and post-nominal adjectives and determiners [81]. Each of the 1404 possible Brocanto2 sentences is meaningful in that it describes a move of a computer-based board game, which provides a context for the subjects to use the artificial language; see Table 1 for an example Brocanto2 sentence, and Figure 1 for an example game board configuration.

**Table 1:** Example correct and word order violation Brocanto2 sentences.

Sentence type	Brocanto2 stimuli					
Correct sentence	*Blom*	*neimo*	*lu*	*neep*	*li*	*praz*
	Blom-piece	square	the	neep-piece	the	switch
	“The square blom-piece switches with the neep-piece.”					
Violation sentence	*Blom*	* *nim*	*lu*	*neep*	*li*	*praz*
	Blom-piece	*capture	the	neep-piece	the	switch
	“The *capture blom-piece switches with the neep-piece.”					

*Note:*

* = violation.

### Procedure

In the original experiment, subjects learned Brocanto2 under either explicit or implicit training conditions (for additional details, see [31], [40]). In the explicit training condition, participants were provided with 13.5 minutes of input of a type similar to that found in traditional grammar-focused classroom settings. Auditorily-presented metalinguistic explanations structured around word categories (e.g., nouns, verbs) were presented along with meaningful Brocanto2 phrases and sentences (which were also auditorily-presented, together with visually-presented corresponding game board configurations). In the implicit training condition, which was designed to represent more implicit language learning contexts and immersion settings, participants received the same amount of training (13.5 minutes), but were exposed only to auditorily-presented Brocanto2 phrases and sentences, together with visually-presented corresponding game boards. All auditory input was pre-recorded. Following training, all subjects underwent practice with the language. Practice, which was identical for the two training groups, consisted of both comprehension and production practice blocks. These alternated every two blocks, with 20 items in each block. For each comprehension item, subjects listened to a pre-recorded sentence in Brocanto2, and were asked to carry out the stated move on the screen using the computer mouse. For each production item, subjects watched a move displayed on the screen and had to describe it with a single oral sentence in Brocanto2. For both types of practice, correct/incorrect feedback was provided, which was identical for the two training groups.

The original study consisted of three experimental sessions. In the first session, subjects were initially given a brief introduction to the computer-based game, and learned the names of the four game tokens (*pleck, neep, blom, vode*) to 100% accuracy (demonstrated by naming each token correctly three times). They then received explicit or implicit training on Brocanto2, followed by practice with the language (see just above). Upon reaching low proficiency (above-chance performance on two consecutive comprehension practice blocks; the explicit and implicit groups did not differ in the number of practice blocks needed to reach low proficiency (*t*(17) = 0.06, *p* = 0.96); over both groups, mean of 6.10 blocks to reach low proficiency) they underwent behavioral and ERP assessment of Brocanto2 (see below). In the second session (1 to 4 days later), participants received the exact same explicit or implicit training as in the first session, again followed by practice, which they continued until they completed a total (over both sessions) of 36 practice blocks. In the third and final session (1 to 5 days after the second session), subjects were first presented with a warm-up of 8 further practice blocks (four comprehension and four production, which, as before, alternated every two blocks, beginning with two comprehension blocks) prior to a second round of behavioral/ERP assessment (“end of training”). Subjects performed at a high level of proficiency by this point: all participants had achieved at least 80% accuracy on comprehension practice, and the average score on the final comprehension practice block in this third test session was above 90% for both groups (explicit: *M* = 0.98, *SD = *0.03; implicit: *M* = 0.92, *SD = *0.14; *t*(17) = 1.27, *p* = 0.22).

The present study, in which subjects returned 3–6 months later (mean of just over 5 months; *M* = 157.5 days, *SD* = 31.6 days, range = 92–206 days), consisted of a single test session. Subjects were again first given a brief introduction to the computer-based game, and again (re-)learned the names of the four game tokens to 100% accuracy. Following this they did not receive any additional explicit and implicit training. Rather, just as in the final test session in the original study, they completed a warm-up of 8 practice blocks prior to behavioral/ERP assessment of Brocanto2 (“retention”). Thus, the amount of warm-up practice was identical prior to the behavioral/ERP assessments at end of training and retention, the two time points contrasted in the present study. As in the third test session in the original study, subjects again reached a high level of proficiency by the end of practice: all participants scored at or above 80% except for one (who scored 75%), and the average score on the final comprehension practice block was at or above 90% for both groups (explicit: *M* = 0.92, *SD = *0.10; implicit: *M* = 0.90, *SD = *0.13; *t*(17) = 0.382, *p* = 0.71).

As discussed above, the 8 practice blocks at the end of training and at retention were designed as brief warm-up sessions, with the expectation that no additional learning would take place. Indeed, at the end of training the 8 practice blocks did not lead to any gain in performance, as evidenced by the finding that performance did not improve significantly between the first and final comprehension practice blocks (first comprehension block at end of training: *M* = 0.92, *SD* = 0.12; final comprehension block at end of training: *M* = 0.95, *SD* = 0.10; no main effects of block (*F*(1,17) = 1.99, *p* = 0.18) or group (*F*(1,17) = 2.01, *p* = 0.18), and no block × group interaction: (*F*(1,17) = 0.09, *p* = 0.76)). This suggests that at this stage of L2 development, 8 practice blocks are not sufficient to lead to additional learning. Furthermore, performance on the final comprehension block at end of training did not differ from the final comprehension block at retention, confirming that the warm-up period prior to retention did not lead to additional learning beyond that evidenced at the end of training (final comprehension block at end of training: see above; final comprehension block at retention: *M* = 0.91, *SD* = 0.11; no main effect of block (*F*(1,17) = 2.73, *p* = 0.12) or group (*F*(1,17) = 0.85, *p* = 0.37), and no block × group interaction (*F*(1,17) = 0.58, *p* = 0.46)). During warm-up practice at retention, there was a significant gain from the first to the last comprehension practice block (first comprehension practice block at retention: *M* = 0.72, *SD* = 0.20; final comprehension block at retention: see above; main effect of block (*F*(1,17) = 19.52, *p*<0.001) but not of group (*F*(1,17) = 0.373, *p* = 0.55), with no block × group interaction (*F*(1,17) = 0.17, *p* = 0.69)). However, because precisely the same amount of practice did *not* lead to learning at end of training, and there was no performance gain between the final comprehension practice blocks at end of training and retention, this improvement does not appear to reflect additional learning. Rather, it may reflect some other process of reactivation or priming of previously learned knowledge – that is, achieving the purpose of the warm-up period. Therefore any ERP changes between the assessments at end of training and retention are unlikely to be explained by further learning during the warm-up period prior to retention.

### Behavioral and ERP Assessment

The behavioral/ERP assessment examined 240 auditorily-presented Brocanto2 sentences, including 40 sentences with a syntactic word-order violation and 40 matched correct control sentences, which constitute the focus of the present study (see Table 1 for examples). Word-order violation sentences were created from each of the 40 correct sentences by replacing a word from one of the five word categories (e.g., noun, adjective, article, verb, adverb) with a word of a different word category that violated the word-order rules of Brocanto2. Thus the correct and violation sentences differed only in this target (correct or violation) word, the onset of which served as the point of comparison for ERP analysis. Violations were equally distributed over (a) the 14 words to the extent possible; (b) the five word categories, with each word category being replaced by each of the other word categories approximately twice (e.g., adjectives were never replaced by articles because that would not yield a word-order violation, and so were replaced by other categories more often); and (c) sentence positions to the extent possible, although violations never occurred on the first word of the sentence. Note that in order for violations to be equally distributed across the word categories, it was necessary for them to occur in the sentence final position when the violation was on the adverb. In all other cases, sentence final violations were avoided. In sum, this balanced design ensured that across trials, the violation and control conditions did not differ with respect to either (i) the critical target words or (ii) the contexts preceding the target words, thus ruling out baseline problems as well as lexical confounds that are often found in previous ERP work on word-order violations (for a discussion see [56]).

Behavioral assessment (acceptability judgment) and ERP recording at retention followed the same protocol as in the original study [31], [40]. Subjects sat in a comfortable chair 70 cm from a 16 inch CRT monitor, in a dark, quiet testing room. Prior to ERP recording, subjects were given instructions and a short practice session, and were asked to minimize eye and body movements during sentence presentation. During ERP data collection, participants were asked to look at a fixation cross that appeared in the center of the screen and remained for the duration of the aural presentation of each Brocanto2 sentence (via ER-4 insert earphones; Etymotic Research, Inc.). Following Friederici et al. [39], sentences were heard one word at a time, with a 50 ms interval of silence between each word, in order to establish acoustically identical baselines and an absence of coarticulation between words, while allowing for relatively natural-sounding sentences. This approach to stimulus presentation minimizes prosodic context effects that may have contributed to previous ERP data [56]. Following the end of the last word of each sentence, the fixation cross remained on the screen for an additional 500 ms, after which time it was replaced by the prompt “Good?” Subjects then had up to 5 seconds to make a judgment about whether the sentence was good or bad, indicated with the buttons of a computer mouse (left for good, right for bad). These acceptability judgment data constituted the dependent measure for behavioral analyses (see below). The next sentence and fixation cross were presented immediately after the response.

Scalp EEG was continuously recorded in DC mode at a sampling rate of 500 Hz from 64 electrodes (extended 10–20 system) mounted in an elastic cap (Electro-Cap International, Inc.), and analyzed using EEProbe software (Advanced Neuro Technology, Enschede, The Netherlands). Scalp electrodes were referenced to the left mastoid, and impedances were kept below 5 kΩ. The vertical electrooculogram (VEOG) was recorded with two electrodes placed above and below the right eye, and the horizontal electrooculogram (HEOG) was recorded with two electrodes placed on the right and left canthi. The EEG was amplified by Neuroscan SynAmps^2^ amplifiers, and filtered on-line with a band-pass filter (DC to 100 Hz, 24-dB/octave attenuation). Off-line, the EEG was re-referenced to the right mastoid and filtered with a 0.16–30 Hz band-pass filter. Data from all target words free of artifacts greater than 40 µV in the electrooculogram and greater than 75 µV in electroencephalogram were included in the analysis.

### Analysis

In order to examine performance for Brocanto2 on the acceptability judgment task at end of training and retention in the explicit and implicit training groups, behavioral responses to the task were first transformed to *d′* scores for each subject. Test session and training group differences in the ability to discriminate correct and violation sentences were then examined by submitting the *d′* scores to a 2×2 ANOVA with Test Session (end of training, retention) as a repeated factor, and Group (explicit, implicit) as a between-subjects factor.

For ERP analysis, EEG data time-locked to the onset of the violation or matched control target word were averaged for each subject for an array of 42 lateral electrodes, using a 200 ms pre-stimulus baseline. These electrodes covered seven levels of anterior/posterior distribution: FP3, FF3, FF1, FF2, FF4, FP4 (anterior-0); F7, F5, F3, F4, F6, F8 (anterior-1); FC7, FC5, FC3, FC4, FC6, FC8 (anterior-2); T3, C5, C3, C4, C6, T4 (central-1); CT7, CT5, CP3, CP4, CT6, CT8 (central-2); T5, P5, P3, P4, P6, T6 (posterior-1); and OL, PO3, O1, O2, PO4, OR (posterior-2). Within each of these levels, the electrodes also covered two levels of hemisphere (right, left), and three levels of laterality. Additionally, 3 midline electrodes (Fz, Cz, POz) were analyzed. Artifact-free target words were analyzed regardless of whether subjects' online judgments were correct or not. Individual ERPs were entered into separate grand ERP averages for the explicitly and implicitly trained groups. Time-windows were selected on the basis of previous research and visual inspection of the grand averages: 150–300 ms for examining possible very early anterior negativities (often referred to as “ELANs” in the literature), 300–500 ms for the N400 and early anterior negativities, and 500–700 as well as 700–900 ms and 900–1200 ms for the P600 and later anterior negativities.

Mean amplitudes for each time window were analyzed using a global ANOVA with the between-subject factor Group (explicit, implicit), the within-subject factors Test Session (end of training, retention) and Violation (correct, violation), and the distributional factors Anterior/Posterior (anterior-0, anterior-1, anterior-2 central-1, central-2, posterior-1, posterior-2), Hemisphere (right, left), and Laterality (from most lateral to medial: lateral-2, lateral-1, medial). When evaluating the Anterior/Posterior and Laterality factors (each of which includes more than one degree of freedom), the Greenhouse-Geisser correction was applied; corrected *p* values are reported. In all cases, any global ANOVA that yielded any significant (*p*<.05) interaction that included the factor Violation was followed up with step-down ANOVAs in order to clarify the nature of the interaction. Analogous analyses were also carried out on the midline electrodes, but without the factors Laterality and Hemisphere. We report significant (*p*<.05) Violation main effects and interactions with Violation from each global ANOVA, as well as lower-level test session, group-specific, or distributional Violation effects revealed by significant step-down analyses. Results of the midline analysis are reported only when they yielded effects that were not evidenced in the lateral analyses.

## Results

### Behavioral

**Figure 2:**
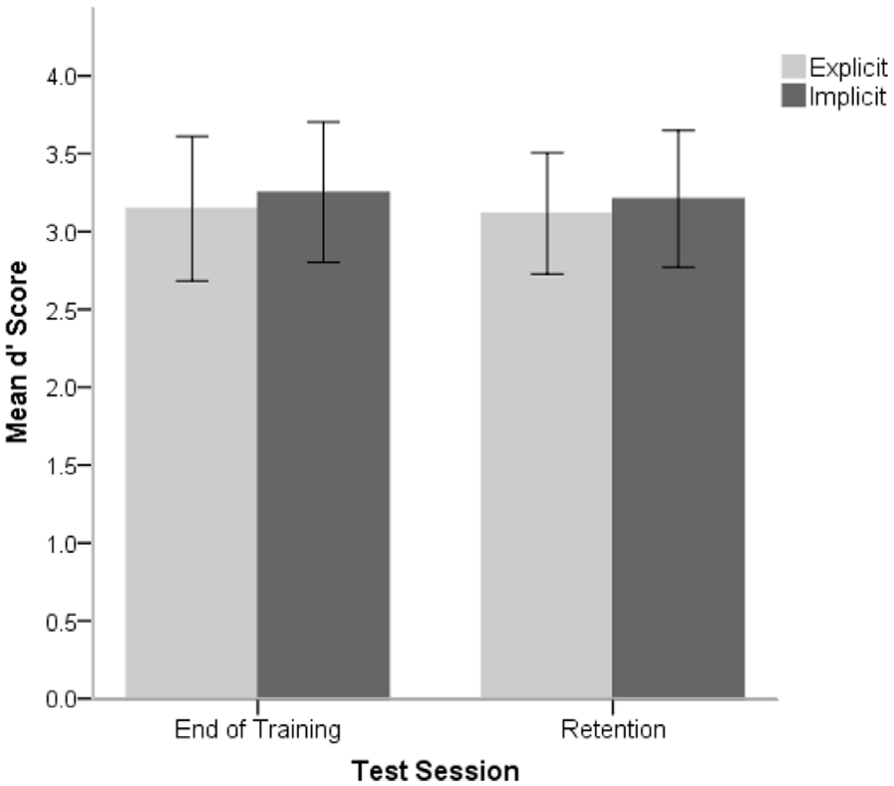
Behavioral results. Mean *d′* scores and standard errors for the explicitly trained and implicitly trained subject groups at end of training and at retention.

The ANOVA between Test Session (end of training, retention) and Group (explicit, implicit) on *d′* scores revealed no main effect of Test Session (*F*(1,17) = 0.04, *p* = 0.86), no main effect of Group (*F*(1,17) = 0.03, *p* = 0.87), and no Test Session x Group interaction (*F*(1,17) = 0.001, *p* = 0.98). The results indicate that at retention both groups retained the high level of proficiency that they had achieved at the end of the original experiment. Indeed, the *d′* scores of both groups in both test sessions were well above 2.5, which corresponds roughly to a proportion correct of 0.90 [82], underscoring the finding that both groups had reached a high level of proficiency (see Figure 2). Finally, as can be seen in Figure 2, and as attested by the ANOVA results, the explicit and the implicit groups performed at similar levels both at end of training and at retention.

### Event-Related Potentials

**Figure 3:**
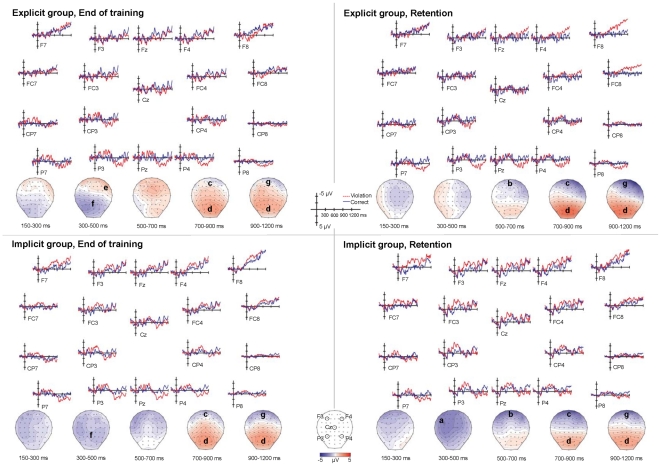
ERP results. Voltage maps and waveforms reflecting the difference between violation sentence and correct sentence grand average ERPs by test session (end of training, retention) and group (explicit, implicit). Significant effects are indicated by letter on the voltage maps. Note that effects (a) through (f) parallel effects (a) through (f) in the Discussion section “ERPs were more native-like at retention than at end of training”. (a) Left anterior-central negativity in the 300–500 ms time window found only at retention in the implicit group. (b) Anterior negativity found over both groups in the 500–700 ms time window only at retention. (c) Anterior negativity found over both groups in both test sessions in the 700–900 ms time window, but which was more robust at retention than at end of training. (d) Posterior positivity found over both groups and both test sessions in the 700–900 ms and 900–1200 ms time windows, but which was more robust at posterior sites at retention than at end training. (e) Right anterior positivity found in the 300–500 ms time window only in the explicit group at end of training. (f) Posterior negativity found over both groups in the 300–500 ms time window only at end of training. (g) Anterior negativity found over both groups and both test sessions in the 900–1200 ms time window.

In the 150–300 ms time window (see Figure 3 for waveforms and voltage maps), the global ANOVA on lateral electrodes elicited four significant interactions with the factor Violation (and no main effect of Violation). Three of these interactions (Violation × Laterality: *F*(2,34) = 9.55, *p* = 0.004; Violation × Test Session: *F*(1,17) = 4.89, *p* = 0.04; and Violation × Test Session × Hemisphere × Laterality: *F*(2,34) = 4.57, *p* = 0.02) were qualified by the five-way Violation × Test Session × Group × Hemisphere × Laterality interaction (*F*(2,34) = 5.55, *p* = 0.01). However, step-down analyses based on this interaction yielded non-significant results.

In the 300–500 ms time window (Figure 3), the global ANOVA on lateral electrodes produced six interactions. Four of these (Violation × Laterality: *F*(2,34) = 9.32, *p* = 0.005; Violation × Test Session × Anterior/Posterior: *F*(6,102) = 7.89, *p* = 0.003; Violation × Test Session × Group × Hemisphere: *F*(1,17) = 9.53, *p* = 0.006; Violation × Test Session × Hemisphere × Laterality: *F*(2,34) = 4.96, *p* = 0.02) were qualified by two higher level interactions: the four-way Violation × Test Session × Anterior/Posterior × Laterality interaction (*F*(12,204) = 2.88, *p* = 0.02) and the five-way interaction among Violation × Test Session × Group × Hemisphere × Laterality (*F*(2,34) = 5.75, *p* = 0.01).

The step-down analyses for the first of these higher-level interactions (Violation × Test Session × Anterior/Posterior × Laterality) revealed three effects over both training groups. At end of training there was both a posterior negativity (posterior-1: medial, *F*(1,17) = 5.17, *p* = 0.04; posterior-2: both medial, *F*(1,17) = 5.55, *p* = 0.03 and lateral-2, *F*(1,17) = 5.34, *p* = 0.03), and a lateral anterior positivity (anterior-0: lateral-2, *F*(1,17) = 6.33, *p* = 0.02). Additionally, at retention, that is, after the period of no exposure, there was an anterior-central negativity (anterior-2: both medial, *F*(1,17) = 6.81, *p* = 0.01 and lateral-1, *F*(1,17) = 4.76, *p* = 0.04; central-1: both medial, *F*(1,17) = 6.45, *p* = 0.02 and lateral-1, *F*(1,17) = 5.08, *p* = 0.03; central-2: medial, *F*(1,17) = 4.87, *p* = 0.04).

Two of these effects were further characterized by the five-way Violation × Test Session × Group × Hemisphere × Laterality interaction and its step-down analyses. First, at end of training, the lateral anterior positivity was revealed to be right lateralized, and only present in the explicit group (Explicit group: right: lateral-2, *F*(1,9) = 9.68, *p* = 0.01). Second, at retention, the anterior-central negativity was revealed to be left lateralized and medially distributed, and only present in the implicit group (Implicit group: left: medial, *F*(1,8) = 5.43, *p* = 0.04).

Thus, for the earlier time windows, specifically for 300–500 ms, the results reveal (1) a posterior negativity found over both groups at end of training but not at retention (Figure 3, effect f); (2) a right lateral anterior positivity for the explicit group at end of training that was not present at retention (Figure 3, effect e); and (3) a left medial anterior-central negativity for the implicit group at retention that was not present at end of training (Figure 3, effect a).

In the 500–700 ms time window (Figure 3), the global ANOVA on lateral electrodes yielded two interactions: a two-way Violation × Anterior/Posterior interaction (*F*(6,102) = 5.52, *p* = 0.01) that was qualified by a three-way Violation × Test Session × Anterior/Posterior interaction (*F*(6,102) = 8.60, *p* = 0.001). Step-down analyses from this three-way interaction revealed an anterior negativity over both training groups only at retention (anterior-0: *F*(1,17) = 8.12, *p* = 0.01; anterior-1: *F*(1,17) = 7.64, *p* = 0.01).

In the 700–900 ms time window (Figure 3), the global ANOVA on lateral electrodes elicited four significant interactions. Three of these (Violation × Laterality: *F*(2,34) = 6.42, *p* = 0.02; Violation × Anterior/Posterior: *F*(6,102) = 17.82, *p* = 0.0002; Violation × Test Session × Anterior/Posterior: *F*(6,102) = 6.03, *p* = 0.009) were qualified by the four-way interaction Violation × Test Session × Anterior/Posterior × Laterality (*F*(12,204) = 3.31, *p* = 0.01). Step-down analyses from this interaction revealed the following effects, all shared by the two training groups. First, an anterior negativity was already present at end of training (anterior-1: lateral-2, *F*(1,17) = 5.51, *p* = 0.03), though it became more robust with a broader distribution at retention (anterior-0: including medial, *F*(1,17) = 7.90, *p* = 0.01, lateral-1, *F*(1,17) = 8.28, *p* = 0.01 and lateral-2, *F*(1,17) = 12.47, *p* = 0.002; anterior-1: including medial, *F*(1,17) = 4.91, *p* = 0.04, lateral-1, *F*(1,17) = 7.70, *p* = 0.01 and lateral-2, *F*(1,17) = 8.41, *p* = 0.01). Second, a P600 was present at end of training (central-2: medial, *F*(1,17) = 4.76, *p* = 0.04; posterior-1: both medial, *F*(1,17) = 11.80, *p* = 0.003 and lateral-1, *F*(1,17) = 10.22, *p* = 0.005; posterior-2: including medial, *F*(1,17) = 12.03, *p* = 0.002, lateral-1, *F*(1,17) = 7.21, *p* = 0.01 and lateral-2, *F*(1,17) = 7.46, *p* = 0.01), but had a more posterior distribution at retention (posterior-1: including medial, *F*(1,17) = 6.80, *p* = 0.01, lateral-1, *F*(1,17) = 6.91, *p* = 0.01, and lateral-2, *F*(1,17) = 8.09, *p* = 0.01; posterior-2: including medial, *F*(1,17) = 13.52, *p* = 0.001, lateral-1, *F*(1,17) = 13.25, *p* = 0.002, and lateral-2: *F*(1,17) = 13.47, *p* = 0.001). Additionally, the global ANOVA on the midline electrodes elicited two interactions: a Violation × Anterior/Posterior interaction (*F*(2,34) = 14.87, *p* = 0.0007) that was qualified by a Violation × Test Session × Anterior/Posterior interaction (*F*(2,34) = 9.95, *p* = 0.0005). Step-down analyses from this latter interaction revealed a P600, shared by the two training groups, which was present at end of training (posterior: *F*(1,17) = 11.18, *p* = 0.004), but was more robust at retention (posterior: *F*(1,17) = 14.27, *p* = 0.002).

In the 900–1200 ms time window (Figure 3), the global ANOVA on lateral electrodes produced two interactions: first, a two-way Violation × Laterality interaction (*F*(2,34) = 11.06, *p* = 0.003), for which step-down analyses yielded no significant results; and second, a two-way Violation × Anterior/Posterior interaction (*F*(6,102) = 22.24, *p*<0.0001). Step-down analyses from this latter interaction revealed both an anterior negativity (anterior-0: *F*(1,17) = 9.47, *p* = 0.006; anterior-1: *F*(1,17) = 6.99, *p* = 0.01) and a P600 (central-2: *F*(1,17) = 5.24, *p* = 0.03; posterior-1: *F*(1,17) = 15.58, *p* = 0.001; posterior-2: *F*(1,17) = 16.90, *p* = 0.001), both of which were found over both training groups and both test sessions. Additionally, the global ANOVA on the midline electrodes elicited the same two interactions as the analogous midline ANOVA in the previous time window (700–900 ms): a Violation × Anterior/Posterior interaction (*F*(2,34) = 18.88, *p* = 0.0002), which was qualified by a Violation × Test Session × Anterior/Posterior interaction (*F*(2,34) = 4.12, *p* = 0.04). As in the previous time window, step-down analyses from this latter interaction revealed a P600 over the two training groups, which was present at end of training (posterior: *F*(1,17) = 6.66, *p* = 0.02), but became more robust at retention (posterior: *F*(1,17) = 13.23, *p* = 0.002).

In sum, for these later time windows (500–700 ms, 700–900 ms, 900–1200 ms), both an anterior negativity and a P600 were evident over both groups and to at least some extent in both test sessions. However, analyses revealed important differences between the test sessions. Over both groups, the anterior negativity appeared earlier, was more robust, and had a broader distribution at retention than at end of training. Specifically, in the 500–700 ms time window it was present only at retention (Figure 3, effect b), and not at end of training, while in the following time window (700–900 ms) it displayed a more robust effect (larger F values) and was more broadly distributed at retention than at end of training (Figure 3, effect c). Only by the 900–1200 ms time window was the anterior negativity statistically equivalent between end of training and retention (Figure 3, effect g). The P600 was present in both the 700–900 ms and 900–1200 ms time windows over both groups in both test sessions (Figure 3, effect d), but showed a more posterior distribution in the 700–900 ms time window at retention than at end of training, and was more robust (larger F values) in posterior sites in both time windows at retention than at end of training.

## Discussion

### Summary

In summary, in this longitudinal (within-subjects) study, healthy adult monolinguals learned an artificial language (Brocanto2) to high proficiency under either explicit, classroom-like, or implicit, immersion-like training conditions, and then underwent several months (mean of about 5 months) of no exposure to the language. Behavioral (acceptability judgment) and ERP data were collected, following brief warm-up periods, both immediately after training (end of training) and after the period of no exposure (retention) in response to Brocanto2 sentences, which were either correct or contained a syntactic word order violation. Although subjects' acceptability judgments did not differ between the end of training and retention, or between the explicit and implicit training groups, ERPs showed striking differences. Here we discuss the behavioral findings and then the ERP results, after which we discuss broader impacts and future directions of the full set of findings, followed by a brief conclusion.

### Behavioral Findings

The behavioral findings have various implications. First, the fact that performance on the judgment task did not differ between the two test sessions or training groups indicates that the observed ERP differences cannot be explained by performance differences. (For discussion of the finding that ERPs but not performance differed between the test sessions and training groups, see the section below on ERP findings.)

Second, the finding that performance did not differ between end of training and retention (for either training group) extends the previous literature examining the behavioral consequences of limited or no L2 exposure. As we have seen above under Previous Research, earlier studies have generally reported lower L2 performance, that is, attrition, subsequent to a period of limited or no exposure. However, the literature is still restricted to very few studies, and these have often been subject to various confounds that suggest caution in interpreting the results. In addition, most studies have not examined grammatical outcomes. The present study – whose longitudinal design, lack of L2 exposure during the delay, and warm-up sessions prior to both assessments addresses some concerns from previous studies – suggests that at least in certain circumstances attrition does not seem to occur. In particular, in this experimental paradigm, a several month period of no exposure following the attainment of high proficiency does not appear to lead to any loss of performance on a measure of grammar. This finding strengthens previous observations that the attainment of high proficiency may reduce [17], [18], [21], [23] or even eliminate attrition [21], and suggests the possibility that grammatical performance might be particularly resilient to attrition after high proficiency has been reached. More generally, the absence of any attrition in the present study suggests the possibility that L2 attrition might not be as common as has previously been reported. In particular, the inclusion of a warm-up period here, as well as in the one previous study that did include some warm-up, and which found no changes and even gains in performance [24], suggests that addressing the confound of recency of L2 exposure may have a significant impact on the outcome of studies of limited or no L2 exposure.

Third, the lack of performance differences between the explicitly and implicitly trained groups both at end of training and at retention at first blush does not appear to be consistent with the previous L2 training literature. This literature has suggested that explicit training generally leads to better performance outcomes than implicit training, even after a delay [28], [29]. However, as discussed above (see Previous Research), earlier studies did not examine subjects at high L2 proficiency, appeared to favor explicit treatments, and were not designed to test the impact of a period of no L2 exposure (e.g., subjects often had contact with the L2 during the delay). Thus, earlier investigations do not seem to be directly comparable to the present study, and therefore their results cannot be taken as inconsistent. Rather, the present experiment extends the literature in important ways, being the first to examine the impact of a substantial period of no exposure following the attainment of high proficiency with either explicit or implicit training. The findings suggest that in these circumstances neither explicit nor implicit training yields a clear advantage at aspects of grammar, at least when measured with a judgment task, either prior or subsequent to the delay. This result complements the findings of the original study, which also reported (with a larger number of participants) a lack of performance differences on the judgment task between the explicit and implicit groups at end of training [31].

### ERP Findings

As we have seen, unlike the behavioral findings, ERPs showed differences both between test sessions and between groups. Importantly, the observed patterns suggest particular and systematic differences in the neural processing between end of training and retention, as well as between the explicit and implicit groups.

### ERPs were more native-like at retention than at end of training

Multiple lines of evidence in this study suggest that both training groups showed more native-like neural processing at retention than at end of training (the following letters (a)–(f) correspond to effects a–f in Figure 3; note that effect g, which does not show test session or group differences, is discussed in various places below, including under (c) in this section): (a) In the 300–500 ms time window, the implicit group elicited a left anterior-central negativity, consistent with native speaker ERP responses to word order and other syntactic violations (see ERP section in Introduction), at retention but not at end of training. (b) In the 500–700 ms time window, an anterior negativity consistent with later negativities found for syntactic processing in native speakers was observed over both groups at retention, but was not present at end of training. (c) In the 700–900 ms time window the anterior negativity, which was present over both groups in both test sessions, was more robust at retention than at end of training; only by the 900–1200 ms time window were there no differences in the anterior negativity between end of training and retention (Figure 3, effect g). (d) In both the 700–900 ms and 900–1200 ms time windows the P600, which is also typical of native-like syntactic processing, was more robust at posterior sites at retention than at end of training (note that the finding that the P600 was less robust at more central sites at retention than end of training in the 700–900 ms time window is consistent with additivity effects from the anterior negativity [56], which was more robust and more broadly distributed at retention than at end of training). (e) In the 300–500 ms time window the explicit group showed a right anterior positivity, which is *not* typical of native syntactic processing, at end of training, whereas this effect was not present at retention. (f) Finally, also in the 300–500 ms time window, over both training groups analyses revealed a posterior negativity, which is again not typical of native syntactic processing, at end of training but not at retention.

The finding that ERPs were more native-like at retention than at end of training suggests the following specific processing changes between the two test sessions. (a) The presence of a left-lateralized anterior-central negativity for the implicit group at retention (Figure 3, effect a) but not at end of training suggests that in the implicit group syntactic processing depended more on rule-governed structure-building [50]–[52], and possibly the procedural memory brain system [10], [53], at retention as compared to end of training. Note that in the original study, Morgan-Short et al. [31] reported a bilateral anterior-central negativity for the implicit group at end of training. The absence of any such effect in the analyses reported here is likely due to lower power from fewer subjects, as well as to a more fine-grained time window in the present study (300–500 ms, versus 350–700 ms in the original study). Importantly, the finding here of an anterior-central negativity in the implicit group at retention but not at end of training suggests that any such effect at end of training is indeed less robust than at retention. Moreover, the fact that the anterior-central negativity was bilaterally distributed at the end of training in the original study, but was left-lateralized at retention in the present study, suggests the greater left-lateralization of any such effect at retention than at end of training. This is a potentially important result, since increased left lateralization has been associated with higher proficiency in L1 [49] (indeed with word order violations in aurally presented sentences, as in the present study), and possibly in L2 [5]. Thus the neural processing of the implicit group at retention was more similar to that of high proficiency native speakers than at end of training. Additionally, the association of left lateralized anterior negativities with higher proficiency in previous studies suggests the possibility that in the present study the use of different performance measures might indeed have revealed higher proficiency at retention than at end of training for the implicit group, or that such proficiency differences might have emerged with further time or practice. Future research may shed light on this issue.

(b) The presence of an anterior negativity over both groups at retention (Figure 3, effect b) but not at end of training in the 500–700 ms time window can be taken to suggest the following. If the anterior negativity in this time window reflects a continuation of the earlier negativity [49], [56], the findings would suggest that rule-governed structure-building, and possibly an increased dependence on procedural memory, took place in this time window for both training groups at retention, but not or less so at end of training. Likewise, if the anterior negativity is involved in increased working memory demands during syntactic processing [54], the findings would suggest that these native-like processes are occurring in both groups at retention but not at end of training. Importantly, whatever the particular mechanistic explanation of this later anterior negativity, the results suggest that both groups show more native-like processing at retention than at end of training in this time window.

(c) The finding of more robust anterior negativities over both groups at retention than at end of training in the 700–900 ms time window (Figure 3, effect c) strengthens the conclusion that both groups rely more on those aspects of native-like processing represented by the later anterior negativity at retention than at end of training. The finding that in the 700–900 ms time window the anterior negativity is present not only at retention, but also for the first time at end of training, suggests that by this time window both groups are depending on such native-like processing not only at retention, but also, even if less so, at end of training. The fact that no test session differences were found (in either group) for the anterior negativity in the 900–1200 ms time window (Figure 3, effect g) indicates that by this time window the two groups do not differ for those aspects of processing represented by the anterior negativity (though they do differ in this time window with respect to the P600; see just below). The earlier onset of anterior negativities at retention (for both groups at 500–700 ms, and for the implicit group at 300–500 ms) than at end of training (for both groups at 700–900 ms, and not equivalent to retention until 900–1200 ms) suggests earlier, perhaps more automatic native-like processing at retention than at end of training (see below for further discussion).

(d) The presence of more robust posterior P600s over both groups at retention than at end of training in the 700–900 ms and 900–1200 ms time windows (Figure 3, effect d) suggests greater native-like controlled processing related to functions such as syntactic integration or structural reanalysis, at retention than at end of training, for both groups.

(e) It is not entirely clear why the 300–500 ms anterior positivity in the explicit group is found only at end of training (Figure 3, effect e), since the processes this effect reflects are not well understood. However, the effect has been interpreted as a possible P3a [31], which underlies attentional mechanisms [83]. The positivity may therefore reflect the use of explicit knowledge, since explicit training conditions are more effective than implicit training conditions in directing learners' attention to L2 forms [33], [84]. Its absence at retention may thus suggest that the explicit group relied at this point less on attentional mechanisms related to explicit knowledge, and more on native-like language processes.

(f) Visual inspection of the voltage maps and waveforms suggests that the posterior negativity found in the 300–500 ms time window over both training groups at end of training (Figure 3, effect f) but not at retention may reflect separate components in the two training groups: an N400 in the explicit group (note the centro-parietal distribution; see Figure 3) and the beginning of the anterior-central negativity in the implicit group (note the extension to frontal electrodes in Figure 3, and the continuing negativity in subsequent time windows, which was significant in the original study; see above). An N400 for the explicit group at end of training seems surprising at first, given that this effect was not reported in the original paper [31]. The difference is probably explained by the selected time windows, since the effect clearly does not extend to the 500–700 ms time window (Figure 3), which was included in the 350–700 ms time window reported in the original paper. The apparent N400 suggests that at end of training the explicit group likely depended on lexical/semantic processing for aspects of syntax, and possibly on the declarative memory brain system [10], [42], [43]. This is particularly intriguing given that in the original study the *implicit* group showed an N400 at low proficiency, but an anterior-central negativity at high proficiency. Thus the explicit group may show a similar trajectory of changes in neural processing over time as the implicit group, but at a greatly delayed rate, so that only at high proficiency does the explicit group show an N400, which the implicit group already showed at low proficiency. Perhaps even more interestingly, the changes in the explicit group between end of training (apparent N400) and retention (anterior negativity beginning as early as 500–700 ms) suggest that this trajectory continues such that the explicit group at retention does not look so different from the implicit group at end of training. For further discussion see below, under Broader Impacts and Future Directions.

What *mechanisms* might explain the pattern of more native-like ERP waveforms at retention than at end of training? First of all, these changes are clearly *not* due to any L2 exposure during the delay, since Brocanto2 is an artificial language developed by our lab with which the subjects could not have had further contact. (Note that, as discussed in Procedure, within Methods, evidence suggests that the brief warm-up period did not lead to any additional learning.) Second, the changes are also unlikely to be due to motivation (see [24]) during the period of no exposure, since even if the subjects had had contact with the language they would have had no clear motivation to learn it, particularly since they had no knowledge of our plan to test them after a delay. Finally, the changes are unlikely to be explained by general maturation or cognitive development [18], since the subjects were already adults prior to the delay (mean age of 25.6), or to continued academic training [18], since they had already had a mean of 16.7 years of education.

So what might in fact account for the observed changes in neural processing? One possibility is that most of the ERP changes can be explained by changes over time in the underlying knowledge (or access to this knowledge) in declarative and procedural memory, two critical long-term memory systems for acquiring, representing, and retaining new higher-level knowledge [53], [85]–[88]. Broadly speaking, a period of no exposure can lead to two opposite types of changes in long-term memory. On the one hand, knowledge or access to that knowledge can weaken, leading to forgetting [89]–[92]. Such declines appear to be worse in declarative than procedural memory [93], [94]. Not surprisingly, forgetting is also associated with activation changes in the brain, at least for declarative memory [93].

On the other hand, research in (cognitive) neuroscience has revealed that both human and animal learners show improvements on a wide range of tasks subsequent to periods without practice with the task or to exposure to the stimuli [95]–[98]. These gains are generally explained in terms of the offline consolidation of knowledge. Such consolidation has been shown to take place in both the declarative and procedural memory systems [99]–[101]. Consolidation is also associated with changes in the underlying neural correlates [98], including changes in brain activation, which may be found even without co-occurring changes in performance [93], [102]. Sleep appears to play a critical role in consolidation in both memory systems, perhaps particularly for procedural memory [98], [100], [103]. Although most research has investigated consolidation after relatively brief periods of non-exposure (e.g., 12 or 24 hours, often with sleep as a factor), some studies have examined longer periods. These have shown that consolidation gains and brain changes can be found after weeks, months or even years [93], [94], [101], [102], [104]. However, performance gains after longer periods are much more consistent for procedural than declarative memory, which in fact generally shows declines (i.e., forgetting) [93], [94], [100]–[102], [104]. Longer-term gains in procedural memory often seem to be due to the longer-term maintenance of consolidation gains already observed after a relatively short period (e.g., 24 or 48 hours), though improvements over longer periods may also take place [101], [103], [104].

Thus, to the extent that syntactic processing depended on declarative and/or procedural memory at end of training in the present study (see above), one would expect performance or brain changes at retention due to forgetting and/or consolidation. In particular, one might expect a decrease in dependence on declarative memory, due to forgetting (despite any consolidation), and an increase in dependence on procedural memory, due to consolidation (and little forgetting). These changes should result in changes in neural processing, and in corresponding changes to ERP patterns. In contrast, changes in performance are more difficult to predict; for example, performance might not change much or at all following a shift in dependence from declarative to procedural memory, since the latter system could take up the slack of the former.

The ERP data appear to be consistent with such underlying changes in declarative and procedural memory. First, evidence suggests a decrease in reliance on declarative memory from end of training to retention. Specifically, at end of training but not at retention the explicit group showed a P3a and an apparent N400, both of which are linked to the declarative memory system: as we have seen above, it has been suggested that N400s depends directly on this memory system, while the P3a may reflect attentional mechanisms related to explicit knowledge, which in turn relies on declarative memory [85]. Thus the disappearance of these two components at retention is consistent with forgetting the underlying knowledge in declarative memory.

Second, the finding that anterior negativities are more reliable at retention than at end of training may be explained by changes in both memory systems. As we have seen, anterior negativities may depend on procedural memory, at least in the 300–500 ms range [10], [53], and also in later time windows if these negativities reflect a continuation of the same processes. Therefore the increased presence of anterior negativities at retention as compared to end of training is consistent with an increased dependence on procedural memory, as would be expected subsequent to the consolidation and strengthening of the underlying procedural knowledge. Such increased dependence on procedural memory at retention seems to hold most clearly for the implicit group, as reflected in the more reliable anterior negativities at 300–500 ms as well as in later time windows, but may also apply to the explicit group, which evidenced such changes only in the later time windows. Note that a dependence on procedural memory does not preclude ongoing or even later onset processing, since this system does not seem to be restricted to early brief processes [105], [106]. Importantly, a greater dependence on procedural memory may be due not only to consolidation in this memory system, but also to forgetting in declarative memory. Evidence suggests that learning in declarative memory, including from explicit training, can inhibit procedural learning or processing [107]–[109]. Although the neurobiological and computational mechanisms of this inhibition are not yet clear [53], [107], [110], they may be related to the blocking phenomenon observed in language, whereby the retrieval of lexicalized knowledge (thought to rely on declarative memory) blocks the application of grammatical rules (thought to rely on procedural memory) [53], [111]. Similarly, if the explicit group at end of training is relying on declarative memory-based explicit knowledge for sentence processing (e.g., paying attention to whether the input is consistent with the grammatical knowledge that they learned), this could simply take precedence over and block any procedural memory-based processes. More generally, whatever the exact mechanisms, any weakening of memories in declarative memory concomitant to forgetting should decrease such inhibition, thereby leading to a greater reliance on procedural memory, as evidenced by the more reliable anterior negativities at retention. Such an effect would hold most clearly for the explicit group, which seemed to rely on declarative memory at end of training (see above). Note that such inhibition could have obscured any procedural knowledge learned in the explicit group by end of training. Thus, the absence of evidence of any early anterior negativities in the explicit group at end of training does not preclude the possibility that this group had indeed acquired procedural grammatical knowledge, which would subsequently have strengthened during consolidation, leading to the increased anterior negativities at retention.

How about the P600? First of all, the finding that this component was more robust at retention than end of training is not likely to be due to consolidation in procedural memory, since the P600 does not appear to depend on this memory system [10]. In contrast, the P600 may rely at least in part on declarative memory structures [10], and thus the observed changes might be at least partially explained by declarative memory-based consolidation. For example, the type of controlled processing reflected by the P600, such as structural reanalysis, might be facilitated by greater declarative memory-based knowledge of the words or rules of the language. However, such an account does not seem likely, since one might expect that at retention such declarative memory-based knowledge should have weakened due to forgetting, rather than strengthening from consolidation. An alternative account seems at least partially consistent with a proposal put forth by Pakulak and Neville [49] that early detection and processing of violations, as reflected by earlier anterior negativities, might free up later controlled resources, as reflected by more robust P600s. On this view, the finding that both training groups showed earlier-onset anterior negativities at retention than at end of training might lead to the expectation that both groups should show more robust P600s at retention, as was indeed observed. However, such an account does not seem entirely consistent with the fact that at retention the implicit group showed earlier anterior negativities than the explicit group: such a difference should lead to more robust P600s for the implicit than explicit group, whereas no such difference was observed (if anything, the P600 seemed more robust in the explicit group; see Figure 3). Future studies may shed light on this issue.

Finally, if forgetting and consolidation in the two memory systems explain at least some of the brain changes, why were there no corresponding behavioral changes? One possibility, as mentioned above, is that the lack of behavioral changes between end of training and retention might be explained by a shift of reliance from declarative to procedural memory. On this view, performance differences between the two test sessions could be minimal since at retention procedural memory would be doing much of the work that declarative memory was doing at end of training. Indeed, such an explanation seems quite plausible for the explicit group, which appeared to rely on declarative memory at end of training, but on procedural memory at retention. However, this account does not seem particularly convincing for the implicit group, since they showed no ERP evidence of declarative memory involvement at end of training. Instead, because brain changes can reflect L2 development prior to the emergence of behavioral changes [37], [38], the ERPs may simply be picking up evidence for underlying brain changes before they are easily detectable with behavioral measures. Note that such an explanation could also hold for the explicit group as well as the implicit group. According to this account, additional time or training would be expected to lead to performance improvements as well. Finally, as was mentioned above, it is also possible that the acceptability judgments simply might not have captured certain performance changes, and thus, despite the observed null effects (i.e., the lack of performance differences between end of training and retention), other behavioral measures might have revealed performance changes between end of training and retention. Future studies should elucidate these issues.

### ERPs were more native-like for the implicit group than the explicit group

Systematic differences in neural processing were found not only between end of training and retention, but also between the explicit and implicit training groups. In particular, in both test sessions the implicit group showed more native-like ERPs than the explicit group. First, in the 300–500 ms time window at end of training, the implicit group did not show any evidence of the non-native-like right anterior positivity found in the explicit group. Second, in the 300–500 ms time window at retention, the left anterior-central negativity elicited by the implicit group was not present in the explicit group, nor was there even a hint of it in the waveforms or voltage maps (see Figure 3). In fact, the explicit group did not show any evidence of an anterior negativity until the next time window (500–700 ms), and never showed any left-lateralized negativity, in any time window.

These ERP differences between the groups suggest the following underlying processing differences. At end of training, the presence of the 300–500 ms anterior positivity only in the explicit group suggests that only this group relied on non-native-like attentional mechanisms, possibly related to the use of explicit knowledge and declarative memory. At retention, the presence of the 300–500 ms left anterior-central negativity only in the implicit group suggests that at retention the implicit but not the explicit group depended on rule-governed structure building, and possibly procedural memory. The subsequent emergence of an anterior negativity over both groups in the 500–700 ms time window indicates a later and perhaps less automatic onset of these processes for the explicit than implicit group at retention. Moreover, the earlier timing of this effect in the implicit group, in the 300–500 ms time window, is consistent with the timing of native speakers, strengthening the view that the implicit group shows more native-like processing than the explicit group.

What might account for the more native-like ERPs in the implicit than explicit group? The differences are unlikely to be due to pre-existing differences between the two groups of subjects, since, as we have seen above, the groups were matched on multiple factors that could affect the outcomes of interest, including age, education, sex, handedness, and language background. The two groups also did not differ in the number of participants who returned from the original study for testing at retention, or in the number of days between completing the original study and testing at retention. Finally, the explicit and implicit groups were matched on the total training time in the original study, and completed the same amount of practice both in the original study and in the warm-up period prior to testing at retention.

The group differences at end of training are thus likely to be explained by differences in the content of the two training paradigms [31]. In particular, the evidence suggests that implicit, immersion-like training leads to more native-like neural processing than explicit, classroom-like training – at retention as well as at end of training. However, it is not yet clear *why* this might be true. One possibility is that the greater native-like processing in the implicit group was due primarily to the larger number of meaningful phrases and sentences presented in the implicit than explicit training conditions (129 vs. 33). On this view, native-like processing critically depends on the number of meaningful phrases and sentences presented to L2 learners, and not on implicit or explicit training per se. Note however that since both training groups heard 440 sentences during comprehension practice up to end of training, and an additional 80 such sentences during the warm-up practice prior to retention, the total number of exemplars presented to the explicit group (553 = 33 exemplars given to both training groups +440+80 comprehension practice items) was only 15% lower than the total number presented to the implicit group (647 = 33 exemplars given to both training groups +94 exemplars given only to the implicit group +440+80 comprehension practice items). Thus the difference in the total number of exemplars between the two groups is not that large, suggesting that this explanation might not fully explain the findings (for discussion, also see [31]). A second possibility is that at end of training the explicit group's dependence on explicit, declarative memory-based knowledge resulted in the inhibition of the learning or use of procedural knowledge (see above), thus precluding anterior negativities. On this view, explicit training actually *prevents*, or at least slows, the development of native-like processing. Moreover, because learning in declarative memory appears to be faster than in procedural memory [53], [108], [109], such blocking is only aggravated by early explicit instruction. Future studies should elucidate this issue.

The conservation of the implicit group's native-like processing advantage at retention shows that even though such a substantial period of no exposure can augment native-like processing in both training groups, it does not necessarily erase the group differences found already by the end of training. It is unclear at this point whether shorter or longer periods of no exposure might yield different outcomes. For example, at shorter periods the explicit group's declarative knowledge would presumably be even more robust than at retention, while at least some procedural consolidation should have occurred in the implicit group (see above), again leading to processing differences between the groups. And after longer periods any further consolidation or even forgetting in procedural memory would presumably have similar effects across the two groups, maintaining the differences already observed at retention. Future studies examining these issues seem warranted.

Finally, the finding that the implicit group showed no performance advantages over the explicit group either at end of training or retention, even while demonstrating greater native-like processing in both test sessions, is an intriguing result. Indeed, the implicit group also showed more native-like neural processing but no performance advantages in the original study, both at low proficiency and at end of training [31]. The findings strengthen the view that similar proficiency levels, even at high levels of proficiency, can be attained using quite different brain mechanisms and types of processing [11]. Additionally, they suggest that this particular period of no exposure, with a brief warm-up period prior to both test sessions, allows learners to maintain a high level of performance, but does not seem to improve the performance of one group more than the other. As discussed above, the underlying mechanisms leading to the maintenance of performance levels between end of training and retention are still not understood, and further studies should reveal whether shorter or longer periods of no exposure, or indeed further training, might lead to different outcomes.

### Broader Impacts and Future Directions

The results from this study show that a substantial period of no exposure to an adult-learned second language does *not* necessarily lead to lower proficiency (use it or lose it), and in fact can even lead to *increased* native-like neural processing. Moreover, the findings show that this pattern may hold independently of the type of training, that is, independently of whether the learner underwent explicit, classroom-like training or implicit, immersion-like training. Thus the study demonstrates that, at least in certain circumstances, a substantial period with no L2 exposure is not necessarily detrimental, and indeed benefits may even ensue over substantial periods, even when such periods do include any L2 exposure.

In particular, the study suggests that subsequent to learning a small but natural-language-like L2 to a relatively high level of proficiency, a several month period of no exposure leads to the observed behavioral and neural outcomes for aspects of grammar. Future studies should reveal to what extent these findings may generalize to other circumstances, including (i) other types of training and practice; (ii) other periods of limited or no exposure, subsequent to the attainment of other proficiency levels; (iii) using other behavioral and neural measures of grammar as well as of other aspects of language; and (iv) other L2s, including not just full natural languages, but also ones with other characteristics and structural differences with the L1. For example, it may be that the results reported here are due to the limited size of the artificial language. More generally, because the longer-term retention of an L2 is generally an important goal for L2 learners, these issues are critical for understanding second language acquisition. Thus this study may be taken as a starting point for a fascinating and useful research program.

The findings of the study may have significant consequences for our understanding of the factors that contribute to the attainment of native-like neural processing of L2 grammar. Previous research on this topic has largely been restricted to examining whether age of acquisition is the sole or primary factor leading to native-like syntactic brain processing, or whether proficiency can also affect it [39], [63]–[65], [112]. Although the examination of these factors has been a reasonable starting point for investigating this issue, it now appears that the story is more complex. First, in our original study, the implicitly-trained learners showed more native-like brain processing than the explicitly-trained learners at end of training, despite the fact that the two groups did not differ on proficiency measures, or on their ages of acquisition or various other factors [31]. The same result was obtained in the present study at retention, where again the subjects did not differ in proficiency or other factors. This suggests that the *type of exposure*, in particular immersion or immersion-like experience, may be an important factor in attaining native-like syntactic processing in the brain. Second, in the present study both groups of participants showed more native-like processing at retention than end of training, despite the finding that they did not differ in proficiency between the two test sessions. This pattern crucially suggests that *substantial periods of time, even with no L2 exposure*, may contribute to the attainment of native-like syntactic processing. Importantly, previous studies implicating proficiency in the attainment of native-like processing have not attempted to take these two factors into account, and thus may have been subject to confounds. Moreover, other factors have also likely been confounded with proficiency in much of this research, in particular the *amount* of L2 training or exposure, which is quite difficult (but by no means impossible) to tease apart from proficiency. Thus overall, the data suggest that multiple factors are likely to affect the attainment of native-like syntactic processing. These include not only age of acquisition, but also type of exposure and substantial periods of time even without any exposure, and presumably amount of exposure as well. Moreover, given that these factors may have confounded the results of previous studies, the role of proficiency itself has yet to be clarified. Future research that carefully teases apart these (and likely other [113]) factors should elucidate exactly which factors contribute to the attainment of native-like syntactic processing in the brain.

The possibility that forgetting and/or consolidation in declarative and procedural memory may have contributed to the observed outcomes has potentially important implications. First, it suggests that future studies should directly test the hypothesis that these two processes in the two memory systems indeed play roles in periods of minimal of no exposure in L2 development. Crucially, a large literature from both humans and animals across multiple tasks and functions has led to an increasingly deeper understanding of these memory systems and processes at many levels, from computational down to molecular mechanisms [53], [85], [98], [114], [115]. Therefore a wide range of relatively specific predictions can be tested. For example, consolidation in declarative memory may happen quite rapidly, on the order of days or less, whereas forgetting increases with increasing time [93], [100], [102], [116]. Thus, performance gains should be observed from consolidation in this memory system primarily after relatively brief delays, on the order of days or less. Indeed, two studies of word learning, which likely depends on declarative memory [53], [117], found that periods of no exposure of 24 hours [118] or 6–10 days [119] yielded performance improvements. As another example, at least some research suggests that consolidation in procedural memory leads to greater performance improvements for those procedures that were more difficult prior to consolidation [120]. Thus aspects of grammar that are particularly difficult for L2 learners, such as morphosyntax [121], might show particular benefits from substantial periods of time, even in the absence of any L2 exposure. And as a final example, research has revealed pharmacological agents that can affect the functioning of these memory systems, including in consolidation [114], suggesting intriguing lines of investigation for L2.

Second and more generally, the likelihood of a role of the two memory systems in L2 retention strengthens the view that the study of non-language domains and systems in both humans and animals, and of these two memory systems in particular, can shed light on language [53], [122], [123]. Conversely, studies of language can elucidate the workings of other domains and systems, including the two memory systems. For example, the apparent shift between declarative and procedural memory from end of training to retention further strengthens the notion that certain tasks can be learned by either memory system and that reliance often shifts of over time from declarative to procedural memory [53], [108], [109].

Finally, the implication of declarative and procedural memory in this study is consistent with the predictions made by the declarative/procedural model for second language acquisition [10], [11], [124], [125]. This neurocognitive model posits that during L2 learning grammar initially depends largely on declarative memory, but that gradually aspects of grammar are increasingly learned and processed in procedural memory. The original study suggested that the implicitly trained group demonstrated this shift, with an N400 at low proficiency and an anterior-central negativity at end of training [31]. The present study suggests that this ERP pattern also occurs in the explicitly trained group, but that the shift occurs much later, and partly as a consequence of presumed consolidation. Thus both implicitly and explicitly trained L2 learners appear to follow the expected shift from declarative to procedural memory, but at a greatly delayed rate for those undergoing explicit training, possibly due to inhibition of proceduralization by the early acquisition of explicit knowledge in declarative memory. Thus the results both further support and specify the model – though note that the implication of the two memory systems and the shift between them by no means precludes at least some other models (e.g., [126], [127]).

The possibility that explicit training may retard the development of native-like grammatical processing is intriguing, and warrants further examination. It suggests that even though explicit training might provide early advantages, its longer term consequences may not be so beneficial. Importantly, this pattern is consistent with previous findings. First, as discussed above, previous studies of explicit and implicit training in second language acquisition have generally reported advantages for explicit training at lower levels of proficiency, that is, early on in the course of learning. Moreover, in our original study we found a performance interaction between group (explicit vs. implicit) and test session (low proficiency vs. end of training): even though the two groups did not differ from each other in either test session, the increase between test sessions was greater for the implicit than the explicit group, suggesting that implicit training may be better at realizing gains towards the attainment of high proficiency. Additionally, as we have seen above, research from declarative and procedural memory suggest that at least in some cases there is an early dependence on declarative memory, but a gradual shift to procedural memory, perhaps due both to more rapid learning in declarative than procedural memory, and to inhibition of the latter by the former. Since explicit knowledge depends on declarative memory, it is not surprising that explicit training would lead to a greater dependence of grammar on this memory system, and that inhibition would therefore slow the process of proceduralization. Thus overall, the evidence indeed seems to suggest that although explicit training can provide fast early grammar learning, it might slow the attainment of native-like grammatical processing and possibly native-like proficiency as well. This has interesting consequences for second language acquisition and training. If the learner's goal is rapid learning rather than the eventual attainment of high proficiency, explicit training might do the trick. But if native-like attainment is desired, explicit training might be harmful, and it might be better to stick solely or largely with more implicit training approaches, such as immersion. Importantly, note that whereas these predictions should hold for grammar, which can depend on either memory system, they should not apply to lexical knowledge, which appears to depend largely or solely on declarative memory [53], [117], and therefore could presumably benefit more from explicit instruction. Finally, it is important to emphasize that these hypotheses and predictions need to be thoroughly examined, including for other aspects of grammar, before being applied to real-world L2 learning contexts.

The results of this study also shed light on the question of whether increased native-like brain processing of grammar should even be a goal for L2 learners. In particular, greater native-like brain processing would be desirable if it correlates with or leads to higher proficiency, which is of course the performance outcome that L2 learners care about. At this point, the data appear to only partially answer this question. On the one hand, as discussed above, the evidence presented here does not suggest a tight correlation between native-like grammatical processing and proficiency, since differences in the degree of native-like brain processing were seen both between groups and between test sessions without any apparent concomitant differences in proficiency. On the other hand, it seems reasonable that only with native-like processing might one eventually attain native-like proficiency, since presumably native speakers use the best available mechanisms for this critical human function. Moreover, it would not be surprising if proceduralization was associated with better proficiency, since processing in the procedural memory system tends to be automatic, rapid and robust [53], [85]. Additionally, previous studies suggest that brain changes often precede behavioral changes [37], [38], and thus the observed increases in native-like processing might *predict* future performance improvements, with additional time or perhaps training. Alternatively, the changes in brain processing might simply be more stable than any changes in performance. On this view, the observed brain patterns might have been attained only days or weeks after the end of training, at which point performance might have peaked, before forgetting set in. As discussed above, it is also possible that other proficiency measures might have revealed a tighter correlation between the level of native-like processing and the level of proficiency in the present study. Other possibilities may also warrant investigation. For example, perhaps native-like processing only yields clear performance advantages for certain structures (such as long distance dependencies [126], [127]) that cannot be easily dealt with by declarative memory. Thus, the relation between native-like processing and proficiency remains to be further elicited.

This study also further clarifies which ERP characteristics may be indicative of more advanced stages of L2 syntactic development. We have already seen that earlier and later anterior negativities, as well as P600s, are associated with L1 grammatical processing, and that these components can be produced by higher proficiency L2 learners under certain circumstances, including at end of training as compared to low proficiency in the original study that forms the basis of the present one [31]. Additionally, we have seen that evidence suggests that higher proficiency in L1 may be associated with more robust P600s, as well as early anterior negativities that are more left-lateralized, less centrally distributed, and less temporally extended to later time windows [49]. In the present study, there were no proficiency differences between the groups or test sessions. Nevertheless, ERP effects or characteristics that have independently been associated with L1 processing, or higher proficiency within L1, clustered together and tended not to co-occur with ERP effects that are not associated with L1. Thus, the implicit group at retention showed the largest L1-like cluster (early and left-lateralized anterior negativities, as well as later anterior negativities and robust P600s), followed by the explicit group at retention (later anterior negativities and robust P600s), and then the implicit group at end of training (later anterior negativities, and less robust P600s than at retention), and lastly the explicit group at end of training (a later anterior negativity and less robust P600s, as well as a non-native-like anterior positivity and possible N400). This pattern of clustering suggests that certain effects and characteristics may be more indicative of more advanced L2 development. In particular, early and left-lateralized anterior negativities may represent the greatest L2 development, followed by P600s with larger amplitudes, followed in turn by later anterior negativities and P600s with smaller amplitudes. In contrast, the results from the present study do not suggest that less centrally distributed and less temporally extended anterior negativities are associated with greater L2 development. No differences between groups or test sessions were found in the degree of either of these characteristics. Rather, anterior negativities showed the same degree of central extension, or lack thereof, as all other anterior negativities (in both groups or test sessions) in their respective time windows. And the appearance of any anterior negativity in any given time window was always followed by anterior negativities in all subsequent time windows (in both groups and test sessions).

Interestingly, the results suggest that *another* ERP characteristic may also be associated with more advanced L2 syntactic development, that is the *onset* of anterior negativities. Anterior negativities showed their earliest onset in the implicit group at retention (300–500 ms), followed by the explicit group at retention (500–700 ms), followed in turn by the two groups at end of training (700–900 and 900–1200 ms), although visual inspection (Figure 3) and analyses from the original study [31] also suggest a later onset for the explicit than implicit group at end of training. Thus, the onset of anterior negativities clusters with other L1-related ERP characteristics, suggesting that the earlier onset of these negativities may be another indicator of more advanced L2 development. (Note that earlier onset and greater amplitude are related characteristics, since if the amplitude in an earlier time window is smaller than in a later one, the effect might not be statistically observed in the earlier one, and thus it would be deemed to have a later onset.) This finding has interesting implications. First, the pattern of anterior negativities with varying onsets between the groups and test sessions seems more likely to represent different onsets of the same process than completely different processes. This strengthens the view that earlier and later negativities likely represent the same process, and that this holds in L2 as well as in L1 [49], [56]. More to the point of L2 development, it suggests that this process, whatever it represents – whether structure building and a dependence on procedural memory or some other process – may occur increasingly earlier, and possibly more automatically, as L2 development proceeds. Second, if indeed anterior negativities have a later onset at earlier stages of L2 development, this would suggest that the absence of earlier anterior negativities in previous L2 studies, including in studies of low L2 proficiency, could be due simply to a delayed onset of the effect, as was observed in the present study. In fact, in some cases an anterior negativity might not be apparent at all, not because the underlying processes are absent, but because they happen to coincide temporally with those of the P600, which might eliminate the negativity (or vice versa) due to additivity effects. Interestingly, this pattern might also explain the absence of earlier and even later anterior negativities in some studies of L1, if indeed delays of this effect are found in L1 as well – for example, if the delay is correlated with the development of L1 (e.g., as reflected by proficiency). Finally, note that the notion of a delayed anterior negativity in L2 is consistent with the finding of delayed N400s in L2 (see Introduction), and moreover suggests the possibility that these N400 delays might also be correlated with the extent of L2 development. Future studies should elucidate these issues.

### Conclusion

This ERP study of an artificial language examined the behavioral and neural consequences of a substantial period of no exposure to an L2, which is a common scenario in second language learning. The results show that, following the attainment of a relatively high proficiency level in the L2, several months of no exposure to the language does not necessarily lead to a degradation of performance, that is, to attrition. Rather, proficiency can be maintained, and an *increase* in native-like neural processing of syntax can occur. The results demonstrate that substantial periods of no exposure are not necessarily detrimental, and that indeed they can be followed by neural gains, at least under some circumstances. Importantly, this pattern was found whether the learners had undergone explicit, classroom-like training, or implicit, immersion-like training, and thus it appears to hold independently of the type of L2 training. Additionally, the implicitly trained group showed more native-like processing than the explicitly trained group both before and after the period of no exposure, indicating that type of training also affects the attainment of native-like processing. Thus, the attainment of native-like syntactic processing in the brain appears to be affected by substantial periods of time, even with no L2 exposure, as well as by type of exposure, in addition to the previously implicated factors of age of acquisition and proficiency (which itself may have been confounded with periods of no exposure and type of exposure in previous studies). The findings in this study may be at least partly explained by a combination of forgetting and consolidation in declarative and procedural memory, two memory systems on which L2 grammar learning appears to depend. Overall, the study has a wide range of implications, and suggests a research program with potentially important consequences for second language acquisition and related fields.
